# Editorial: Neurobiological underpinnings of neurodegenerative and neuropsychiatric disorders: from models to therapy

**DOI:** 10.3389/fnins.2025.1627262

**Published:** 2025-06-09

**Authors:** Ferdinando Di Cunto, Maria Vincenza Catania, Nicola Simola, Jessica Mingardi, Marcello Serra, Federica Campanelli

**Affiliations:** ^1^Department of Neuroscience 'Rita Levi Montalcini', University of Turin, Turin, Italy; ^2^Institute for Biomedical Research and Innovation, National Research Council (IRIB-CNR), Catania, Italy; ^3^Department of Biomedical Sciences, University of Cagliari, Cagliari, Italy; ^4^School of Medicine and Surgery, University of Milano-Bicocca, Monza, Italy; ^5^Department of Neuroscience, Università Cattolica del Sacro Cuore, Rome, Italy

**Keywords:** brain disease mechanisms, neurodegeneration, animal models, neurodevelopmental disorders, acute brain injury

During the last two decades, seminal advances in different areas of Neurosciences have clearly highlighted the complex nature of neuropsychiatric and neurodegenerative disorders. The proper assembly, maturation, and maintenance of neuronal circuits underlying physiological functioning of the nervous system require the execution of specific genetic programs, finely tuned in space and time.

The widespread detection of genes, mRNAs, proteins, and metabolites has shed much light on relevant missteps in these delicate processes that may cause detrimental consequences for brain wiring and function. A clear role of cell-autonomous alterations, involving cell adhesion molecules, ion channels, and intracellular pathways impinging on synaptic function is well established in neuropsychiatric diseases. Similarly, modifications in protein sequence and structure resulting in the accumulation of insoluble aggregates are widely acknowledged as culprits of most neurodegenerative disorders. On the other hand, the careful study of pathophysiological mechanisms that may translate genetic predisposition into fully manifested disease phenotypes has revealed the involvement of non-genetic and non-neuronal factors in establishment and propagation of the damage from cell to network level. Indeed, current research is emphasizing the crucial role of epigenetic modifications, metabolic rewiring, brain-immune system cross-talk, glial cell functions, gut-to-brain axis, and blood-brain barrier, both in neuropsychiatric and neurodegenerative disorders. Importantly, these additional layers of complexity represent a rich interface between genetic variants and environmental stressors, whose nature and relevance are also being elucidated.

A critical factor in advancing the translation of this knowledge into enhanced diagnostic and therapeutic strategies lies in the accurate modeling of human disease pathogenesis. This necessitates comprehensively considering the complexity of cellular and molecular interactions, as well as species-specific characteristics that drive the disease processes. Traditional cellular and animal models have so far been of utmost importance for understanding the molecular mechanisms underlying brain disorders. However, their actual translational value has also been questioned, due to the failure of several clinical trials resulting from pharmacological findings in animal models. Different cutting-edge strategies, including bioprinting, microfluidics 3D cell co-culture, human organoids, single-cell multi-omics, *in vivo* functional imaging, and multidimensional data integration have great potential to help filling the gap between *in vitro* and *in vivo* molecular-cellular-network responses, as well as to reduce the attrition rate between preclinical research and clinical applications. The complexity of these research tools also poses major challenges in terms of reliability and reproducibility.

This Research Topic, proposed on the occasion of the XX meeting of the Italian Society for Neuroscience (SINS) was aimed at advancing the understanding of state-of-the-art techniques and methodologies for modeling neuropsychiatric and neurodegenerative disorders. Specific focus was on critical aspects of the complex genetic, molecular, and cellular mechanisms underlying these conditions, and on novel avenues in brain disease modeling.

The selection and peer review process resulted in the publication of 10 papers, including four original research contributions and six review or mini-review articles, covering a wide range of congenital and acquired neurological conditions and employing diverse modeling strategies.

## *In vivo* modeling of neurological disorders

The majority of the studies included in the Research Topic are focused on the usage of mouse or zebrafish as disease models.

The research report by Chen Z. et al. explored the lifespan progression of dysfunction, oxidative stress, and neuroinflammation in a mouse model of moderate Traumatic Brain Injury (TBI). The findings reveal persistent cognitive and emotional deficits in middle-aged mice. These behavioral changes were linked to increased neuronal cell death, elevated oxidative stress, and chronic neuroinflammation in the hippocampus, suggesting that persistent oxidative stress and neuroinflammation play a significant role in the long-term neurological decline following TBI, providing potential therapeutic targets for TBI-induced late-phase neurological dysfunction.

The brief report by Tanaka et al. presents a new mouse model carrying the Bassoon (Bsn) p.P3882A mutation, analogous to the human BSN p.P3866A mutation found in a family with progressive supranuclear palsy-like syndrome (PSP-like syndrome). Although no significant structural brain abnormalities were observed, this model exhibits impaired working memory and decreased locomotor activity, providing a valuable tool for investigating the relationship between Bsn mutations, tauopathy, and the development of PSP-like syndrome.

The transgenic mouse model provided in the Research Report by Chen L. et al. is characterized by mTORC1 pathway hyperactivation in striatal inhibitory neurons. This model shows disrupted dopamine receptor expression and behavioral abnormalities such as impaired motor learning and altered olfactory preference. These findings offer a molecular basis for understanding the deficits seen in neurodegenerative disorders such as Parkinson's (PD) and Huntington's diseases, as well as neuropsychiatric disorders such as autism-spectrum disorder (ASD).

The mini review contributed by Bossini and Sessa highlights the importance of using diverse and complementary approaches when developing mouse models for neurological diseases. The authors argue that relying on single-approach models may contribute to the high failure rates of clinical trials by providing incomplete or misleading data. Focusing on neurodevelopmental disorders, they advocate for incorporating strategies such as cell-specific gene manipulation, temporal control of gene expression, and molecular reversibility to create more robust and translatable models.

In their research report, Naderi et al. utilized CRISPR-Cas9 technology in zebrafish larvae to investigate the socio-cognitive consequences of KCC2 disruption, a protein involved in excitatory/inhibitory (E/I) balance. This model demonstrates impaired social interactions and memory deficits, along with molecular changes in GABAergic, glutamatergic, oxytocin, and BDNF systems, highlighting the role of KCC2 in these functions and suggesting potential therapeutic avenues.

The growing utility of zebrafish models is further underscored by the mini-review contributed by Bagwell and Larsen, who discussed the mechanisms of MPTP toxicity and highlighted the advantages of zebrafish for high-throughput screening and their ability to recapitulate biochemical mechanisms and symptoms of human PD, addressing limitations of larval models for age-related conditions.

## Investigating molecular and cellular pathogenesis of neurological disorders

Two papers of the Research Topic delve into the molecular and cellular underpinnings of acquired brain disorders or into the conditions that may prevent them.

In particular, the research report by Yao et al. investigated how a perioperative enriched environment (EE) attenuates postoperative cognitive dysfunction (POCD) in a mouse model of ischemic stroke. The authors found that an EE improved neurological function and reduced neuroinflammation in mice subjected to stroke and surgery. This protective effect is mediated by the upregulation of microglia TREM2, via the PI3K/Akt pathway, underscoring the potential of targeting TREM2 and utilizing EE for managing POCD in stroke patients.

In addition, the review by Cong et al. examines the role of the mitochondrial permeability transition pore (mPTP) in the pathogenesis of Intracerebral Hemorrhage (ICH). The authors discuss how mPTP opening leads to mitochondrial dysfunction and contributes to various pathological processes, including oxidative stress, apoptosis, necrosis, autophagy, and ferroptosis. While ICH is a stroke subtype, the mechanisms discussed are highly relevant to neurodegenerative processes and the authors advocate for further investigation into the role of mPTP as a potential therapeutic target for managing ICH-induced secondary injury.

On the front of neurodegenerative disorders of genetic origin, the review by Cui et al. summarizes the pathogenesis of Spinocerebellar Ataxias (SCAs), discussing how various genetic mutations disrupt cellular processes leading to neuronal dysfunction and loss. Although not a modeling paper, it synthesizes knowledge crucial for designing relevant models and developing therapies for SCAs.

Finally, a systematic review by Tesfaye et al. explores brain functional connectivity changes in hyperthyroid patients, a condition that can cause mood and cognitive impairments. It suggests that altered connectivity, particularly involving the hippocampus, may influence cognitive and emotional processing in the framework of hyperthyroidism.

In conclusion, the accepted manuscripts contributed significantly to the Research Topic by presenting original research using diverse *in vivo* animal models (mice, zebrafish) and genetic tools to study the molecular, cellular, and behavioral aspects of various neurodegenerative and neuropsychiatric disorders. Additionally, review articles synthesized existing knowledge on disease mechanisms, modeling challenges, and therapeutic avenues, reinforcing the topic's goal of advancing modeling and understanding pathogenesis of brain diseases.

